# Long-term patient satisfaction and complication rates after orthognathic surgery: a minimum 3-year follow-up study

**DOI:** 10.1007/s10006-026-01580-2

**Published:** 2026-05-27

**Authors:** Șerban Talpoș, Dan Loloș, Tareq Hajaj, Robert Avramut, Camelia Szuhanek, Bogdan Antonio Loloș, Andreea Raissa Hojda, Sorin Gheorghe Mihali, Daniel Raul Chioibaș, Mălina Popa

**Affiliations:** 1 Discipline of Oral and Maxillo-Facial Surgery, Faculty of Dental Medicine, “Victor Babeș” University of Medicine and Pharmacy Timișoara, Revoluției Boulevard 9, Timișoara, 300041 Romania; 2 Faculty of Dental Medicine, “Victor Babeș” University of Medicine and Pharmacy Timișoara, Eftimie Murgu Square No. 2, Timișoara, 300041 Romania; 3 Discipline of Prostheses Technology and Dental Materials, Faculty of Dental Medicine, “Victor Babeș” University of Medicine and Pharmacy, Eftimie Murgu Square 2, Timișoara, 300041 Romania; 4 Doctoral School, “Victor Babes” University of Medicine and Pharmacy, Eftimie Murgu Square 2, Timisoara, 300041 Romania; 5 Department of Orthodontics, Faculty of Dental Medicine, Victor Babes University of Medicine and Pharmacy, 9 No., Revolutiei 1989 Bv, Timisoara, 300041 Romania; 6 Faculty of Medicine, “Vasile Goldis” Western University of Arad, 94 Revolutiei Blvd, Arad, 310025 Romania; 7 Faculty of Medicine, “Victor Babeș” University of Medicine and Pharmacy Timișoara, Eftimie Murgu Square No. 2, Timișoara, 300041 Romania; 8 Department of Prosthodontics, Faculty of Dentistry, “Vasile Goldiș” Western University of Arad, 94 Revoluției Blvd, Arad, 310025 Romania; 9 Department of Surgery, Emergency Discipline, Victor Babes University of Medicine and Pharmacy, Timisoara, 300041 Romania; 10 Department of Pediatric Dentistry, Faculty of Dental Medicine, “Victor Babeș” University of Medicine and Pharmacy, Eftimie Murgu Square 2, Timișoara, 300041 Romania

**Keywords:** Orthognathic surgery, Patient satisfaction, Dentofacial deformities, Long-term outcomes, Postoperative complications, Quality of life

## Abstract

**Background and objectives:**

Orthognathic surgery aims to correct dentofacial deformities and improve both function and facial esthetics. This study assessed long-term patient satisfaction and complication rates following bimaxillary orthognathic surgery, using a customized questionnaire to evaluate esthetic, functional, and psychosocial outcomes.

**Materials and methods:**

A total of 126 patients who underwent bimaxillary surgery for Class II or III dentofacial deformities were included in this retrospective cohort study with prospectively collected data and evaluated after a minimum follow-up of 3 years. Surgical procedures included Le Fort I osteotomy, bilateral sagittal split osteotomy, multisegmental osteotomies, genioplasty, and SARPE, with all cases planned and managed by an interdisciplinary team. Patient satisfaction was assessed using a 20-item individualized questionnaire based on validated tools (OQLQ, DAS-59, OHIP-14, VAS).

**Results:**

High levels of long-term satisfaction were reported, particularly in smile appearance and self-confidence. Smile-specific satisfaction remained stable regardless of skeletal class, gender, or complications. Postoperative complications occurred in 11.1% of patients and represented the main observed factor significantly associated with reduced overall satisfaction (p < 0.001). The most frequent complications included mandibular plate infection (n = 5), mandibular osteoperiostitis (n = 4), plate detachment (n = 2), plate fracture (n = 1), pseudoarthrosis (n = 1), and malunion with vicious callus (n = 1). All were successfully managed without long-term sequelae.

**Conclusions:**

Orthognathic surgery provides consistent long-term esthetic and psychosocial benefits. Individualized planning, interdisciplinary management, and the use of a customized satisfaction questionnaire may have contributed to the favorable patient-reported outcomes observed. These findings support the role of orthognathic surgery in enhancing quality of life beyond skeletal correction.

**Supplementary Information:**

The online version contains supplementary material available at 10.1007/s10006-026-01580-2.

## Introduction

Orthognathic surgery remains a key solution for correcting skeletal dentofacial deformities, aiming to improve facial balance, occlusal function, and overall quality of life for patients [1, 2]. The advent of virtual surgical planning and CAD/CAM systems has increased procedural accuracy while reducing intraoperative risks [3–5]. This technological progress has coincided with a growing interest in long-term outcomes, particularly regarding patient satisfaction and the prevalence of late complications [6–9]. 

Although functional and aesthetic improvements are generally achieved, some studies still report postoperative concerns such as neurosensory deficits, TMJ symptoms, or reduced patient satisfaction [10–12]. These findings emphasize that outcomes depend not only on surgical precision but also on managing psychological readiness and patient expectations [13, 14]. While many patients report improved self-image and social integration after surgery, conflicting evidence exists about the consistency between objective clinical outcomes and subjective satisfaction [15–19].

Long-term follow-up beyond three years remains limited, although it is essential to evaluate late complications, psychosocial adaptation, and potential retreatments [20–22]. There is increasing recognition that treatment success must extend beyond occlusal and skeletal criteria, integrating patient-centered outcomes such as emotional well-being, perceived facial harmony, and reintegration into social and professional life [18, 19, 22–24]. However, the correlation between surgical success and subjective satisfaction remains controversial, particularly in patients undergoing multiple procedures or those with high aesthetic demands [25, 26].

Functional recovery is also evolving through protocols such as electroacupuncture, which may accelerate neurosensory rehabilitation [27]. Additionally, the integration of segmental osteotomies, guided expansion, and facial contouring has enhanced precision in addressing complex asymmetries [28, 29]. The role of surgical timing has also changed, with surgery-first or early-surgery protocols becoming more popular for select cases. These approaches allow for quicker aesthetic improvements and shorter treatment duration but demand strict case selection and interdisciplinary coordination [30–33].

Despite innovation, treatment protocols still vary widely across institutions. A recent survey revealed minimal consensus on perioperative protocols among academic maxillofacial surgeons, underlining the need for evidence-based standardization [34]. Orthodontic planning remains a cornerstone of success, and modern guidelines stress the continuous involvement of the orthodontist from diagnosis through retention [35].

In addition to occlusal and skeletal considerations, contemporary orthognathic surgery increasingly emphasizes soft tissue balance and facial aesthetics. The concept of the “Barcelona Line,” introduced by Alfaro, has become a valuable cephalometric and visual tool for evaluating midfacial harmony and menton positioning [36, 37]. This method enhances aesthetic planning and guides rotational adjustments of the maxillo-mandibular complex. Distinguishing dentoalveolar malocclusions from true dentofacial deformities (DFDs) remains critical, as the latter require a combined orthodontic-surgical approach [38]. In such cases, interdisciplinary evaluation must precede decompensatory orthodontics, aligning dental arches to skeletal bases and preparing the occlusal plane for surgery [39].

Beyond physical outcomes, orthognathic surgery significantly impacts psychosocial perception. Studies show that dentofacial anomalies affect perceived intelligence, sociability, and emotional stability. Surgical correction often improves these perceptions, enhancing both external evaluations and patients’ internal self-esteem [40–43]. Still, psychological readiness and personality traits influence treatment decisions and postoperative satisfaction. Patients with high aesthetic concern or perfectionistic tendencies may require additional psychological support [44]. Unfortunately, most orthodontists do not routinely refer patients for psychological evaluation, even though such assessments could improve overall outcomes [45]. This need is particularly relevant in surgery-first protocols, where rapid changes may cause emotional distress early in recovery [46].

To evaluate these dimensions comprehensively, our study employed a custom 20-item questionnaire that integrates validated components from the Orthognathic Quality of Life Questionnaire (OQLQ), the Derriford Appearance Scale (DAS-59), the Oral Health Impact Profile (OHIP-14), and Visual Analogue Scales (VAS) [8–49]. This hybrid instrument allowed participants not only to rate satisfaction in esthetic, functional, psychological, and social domains, but also to rank the relative importance of treatment goals from a patient-centered perspective. By using this approach, the present study aims to assess long-term outcomes following orthognathic surgery, focusing on complications, perceived success, and psychosocial adaptation beyond the three-year threshold.

Given the limited data on long-term patient-reported outcomes and the ongoing debate regarding the correlation between surgical success and subjective satisfaction, this study aims to fill a critical gap by evaluating both complication rates and multidimensional patient satisfaction at a minimum of three years after orthognathic surgery, using a customized questionnaire developed from validated instruments to integrate esthetic, functional, psychological, and social domains.

## Materials and methods

This retrospective cohort study with prospectively collected data was conducted at the private dental and surgical clinic Confident by Dr. Talpoș in Timișoara, Romania, where all cases were managed following standardized interdisciplinary protocols. The study adhered to the principles of the Declaration of Helsinki and received ethical approval from the Ethics Committee of the “Victor Babeș” University of Medicine and Pharmacy in Timișoara, Romania (Approval Nr. 105/06.01.2020, revised 2025). Written informed consent was obtained from all patients or, in the case of minors, from their legal guardians.

All patient data, including clinical records, photographic documentation, and questionnaire responses, were collected and organized in a password-protected Microsoft Excel^®^ database, in compliance with GDPR regulations and institutional data protection protocols.

All surgical procedures were performed by the same maxillofacial surgeon to ensure consistency in technique and intraoperative management. Orthodontic treatment for all included patients was carried out by a single orthodontist, who was actively involved in all clinical stages of care, including preoperative planning, intraoperative support, and postoperative evaluations. Interdisciplinary case reviews and treatment decisions were made collaboratively by the surgeon and orthodontist throughout the study period to maintain clinical continuity and uniformity. Individualized treatment planning was considered a critical step, based on the minimally invasive orthognathic surgery concept, and focused on both functional and esthetic objectives such as bite correction, facial balance, TMJ and periodontal health, airway optimization, and long-term stability. This approach aimed to reduce morbidity, minimize inflammatory response, improve postoperative comfort, and enhance esthetic outcomes. Although most patients emphasized esthetic concerns, treatment plans were designed to integrate these expectations within medically sound parameters.

The study was initiated on January 6, 2020, and completed on June 2, 2025, with all patients having a minimum follow-up duration of three years.

All patients underwent a mandatory interdisciplinary consultation with both the maxillofacial surgeon and the orthodontist prior to inclusion in the study. This joint evaluation allowed for precise diagnosis, aesthetic and occlusal analysis, and treatment planning. All photographs were taken under standardized lighting conditions, with the same camera, background, and patient positioning to ensure consistency of pre- and postoperative documentation.

**Fig. 1:**
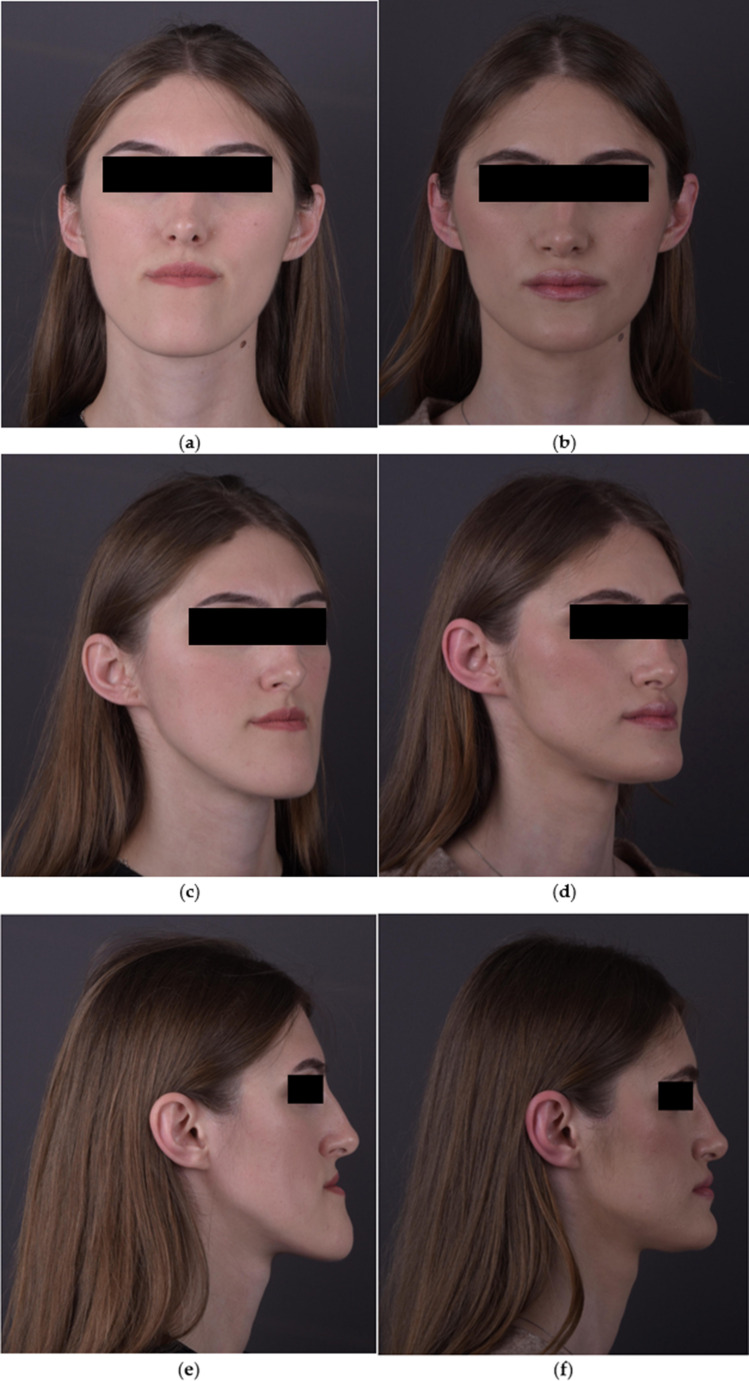
(**a**) Frontal view before orthognathic surgery showing mandibular retrusion and lower facial asymmetry. (**b**) Frontal view one year postoperatively demonstrating improved symmetry and smile aesthetics. (**c**) Right semi-profile view before surgery highlighting convex profile and chin retrusion. (**d**) Right semi-profile view after surgery showing enhanced facial contour and projection. (**e**) Profile view prior to surgery with pronounced skeletal Class II pattern. (**f**) Profile view at one-year post-surgery showing normalized facial profile and improved lower third balance

**Fig. 2:**
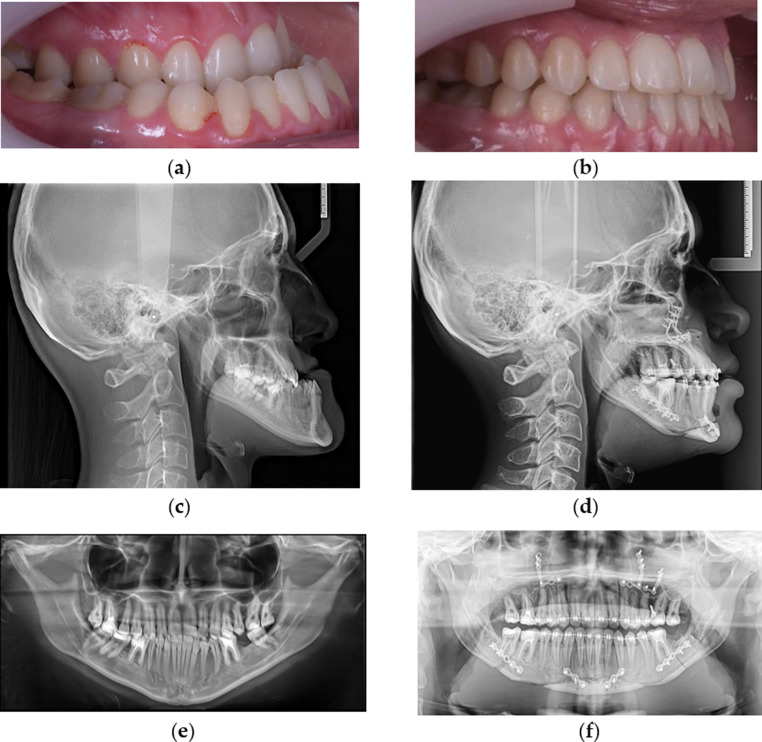
(**a**) Preoperative occlusal view showing Class II molar and canine relationship. (**b**) Postoperative occlusal view at one-year follow-up, indicating improved Class I relationship and overjet correction. (**c**) Initial lateral cephalometric radiograph demonstrating mandibular retrognathism and increased lower facial height. (**d**) Postoperative lateral cephalogram at one year showing improved skeletal profile and occlusal plane alignment. (**e**) Preoperative panoramic radiograph (orthopantomograph) illustrating dental alignment and root morphology. (**f**) One-year postoperative panoramic radiograph revealing stable osteosynthesis with miniplates and screws, corrected occlusal relationship, and root integrity

Aesthetic analysis was supported by photographic documentation in frontal, semi-profile, and profile views (Figs. 1a–f), in addition to occlusal assessments, lateral cephalograms, and orthopantomographs (Fig. 2a–d).

**Fig. 3:**
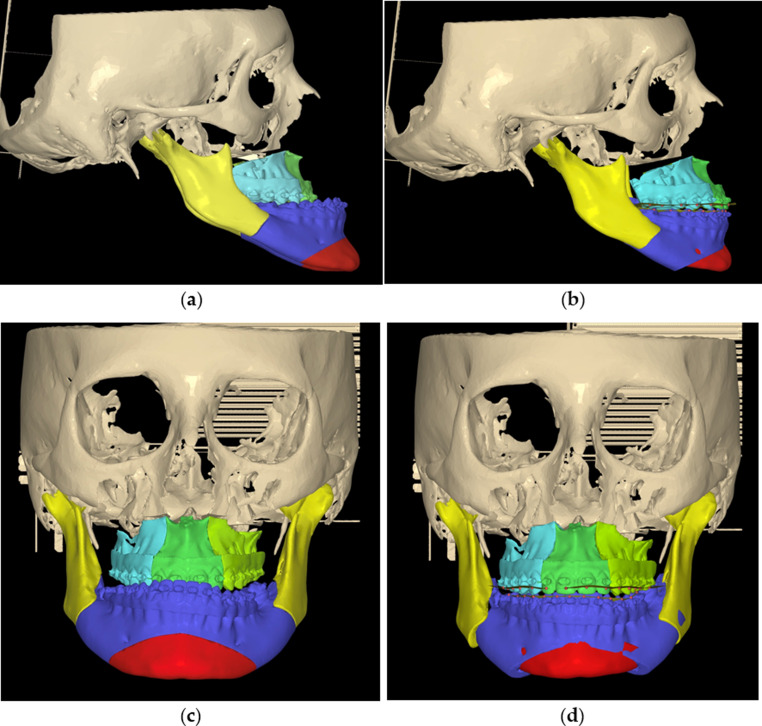
Virtual surgical planning using Nemofab^®^ software (Nemotec, Madrid, Spain): (**a**) Lateral 3D view showing segmentation of the mandible and maxilla before osteotomy simulation. (**b**) Lateral 3D view after simulation of mandibular repositioning with planned osteosynthesis. (**c**) Frontal 3D view illustrating segmentation of skeletal components and osteotomy lines. (**d**) Frontal 3D view after virtual repositioning of maxillary and mandibular segments, showing final occlusal and esthetic alignment

For virtual surgical planning, Nemofab^®^ software (Nemotec, Madrid, Spain) was employed, enabling 3D segmentation of the maxilla and mandible, simulation of osteotomies, and repositioning of skeletal segments as seen in Fig. 3.

**Fig. 4:**
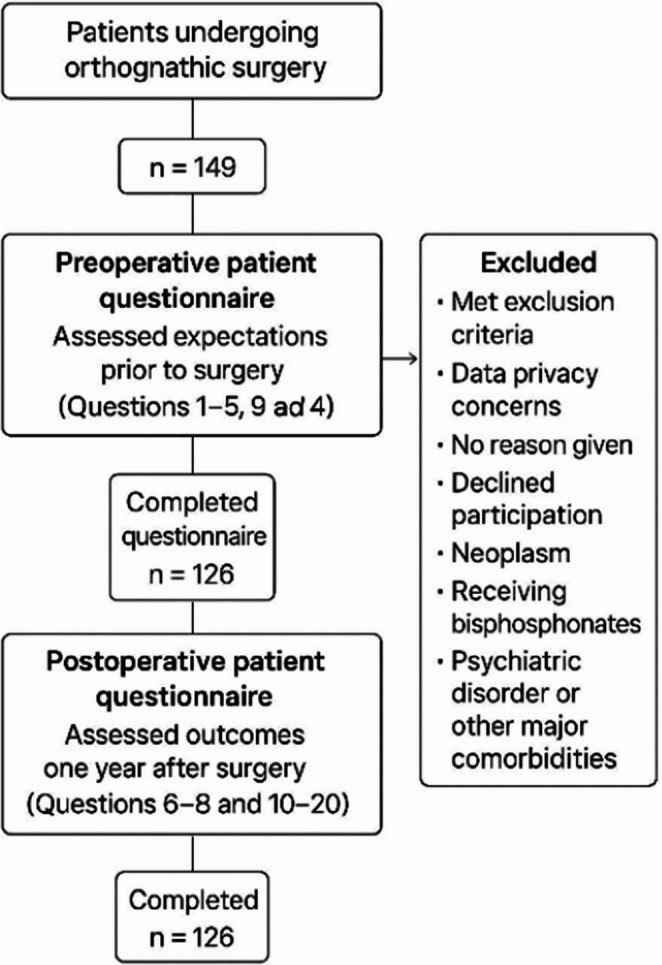
Study flow diagram of patient inclusion and questionnaire completion

Out of the 149 patients initially evaluated, 126 met the inclusion criteria and were enrolled in the final analysis. The study design and patient flow are summarized in Fig. 4.

Exclusion criteria included withdrawal of consent, failure to complete follow-up assessments, deviation from the surgical-orthodontic treatment protocol, congenital syndromes (e.g., Crouzon syndrome, Apert syndrome, Marfan syndrome, cleft lip and palate), neoplasms, bisphosphonate therapy, severe psychiatric disorders, or refusal to consent to data and image use. Additional patients were excluded due to incomplete consent for data processing or lack of authorization for image and data publication. Moreover, prior to treatment, patients exhibiting unrealistic aesthetic expectations or psychological profiles incompatible with the goals of orthognathic surgery were not considered suitable candidates and were not included in the surgical cohort.

All included patients were diagnosed with skeletal Class II or Class III dentofacial deformities. Orthodontic treatment, performed by the same orthodontist for all cases, followed a standardized protocol consisting of an average of 18 months of preoperative decompensation and up to 6 months of postoperative refinement to achieve Class I occlusion. Elastic intermaxillary activations were applied every two days during the first two postoperative weeks, under joint supervision by the orthodontist and surgeon. All patients received retention devices for at least one year after treatment completion.

Female patients were required to be at least 16 years of age, and male patients at least 18 years, with documented completion of craniofacial growth.

**Fig. 5:**
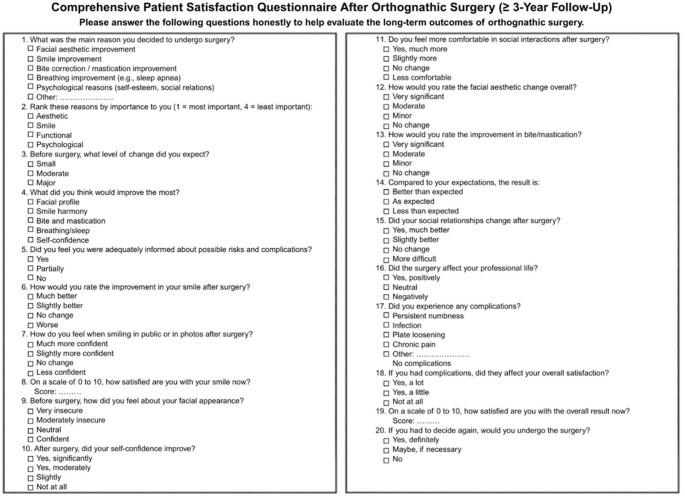
Customized 20-item questionnaire assessing patient expectations and outcomes after orthognathic surgery

A customized 20-item satisfaction questionnaire was developed to capture both preoperative expectations and postoperative outcomes as seen in Fig. 5.

Existing validated questionnaires were considered insufficient to fully capture specific esthetic expectations and patient-prioritized outcomes relevant to orthognathic surgery. Therefore, a customized instrument was developed by integrating key components from established tools with additional items designed to reflect clinically relevant aspects not adequately addressed in the literature. This approach allowed for a more comprehensive, patient-centered evaluation of esthetic, functional, and psychosocial outcomes.

This instrument combined validated elements from the OQLQ, DAS-59, OHIP-14, and Visual Analogue Scales (VAS) with original components created by the authors. It included multiple-choice items, scaled responses (e.g., 0 to 10 satisfaction scores), and ranked prioritizations to evaluate aesthetic, functional, psychological, and social domains. Unlike standard tools, this questionnaire allowed patients not only to quantify perceived outcomes but also to rank the importance of treatment goals.

Preoperative assessment included Questions 1–5, and 9 (focused on expected improvements), while Questions 6–8 and 10–20 were completed exactly one year after surgery to ensure standardized timing of evaluation. Patient-reported satisfaction outcomes were therefore assessed at a fixed one-year postoperative timepoint. Although all participants had a minimum overall follow-up period of three years, this extended follow-up was used to monitor clinical outcomes and complications. The use of a fixed one-year evaluation point was intended to ensure consistency of responses across the cohort and to minimize variability related to differences in follow-up duration.

The questionnaire was pilot-tested in 10 patients to assess clarity, internal consistency, and clinical applicability.

Surgical interventions were standardized and included Le Fort I osteotomy of the maxilla, bilateral sagittal split osteotomy (BSSO) of the mandible, multisegmental osteotomies in cases of maxillary transverse deficiency < 8 mm, and adjunctive genioplasty for advancement or setback where indicated. In cases exceeding 8 mm of transverse deficiency, SARPE (surgically assisted rapid palatal expansion) and palatal expansion devices were applied preoperatively.

Rotational adjustments of the maxillo-mandibular complex, particularly counterclockwise rotation, were performed in many Class II cases. Genioplasty planning followed both cephalometric measurements and aesthetic considerations, including the Barcelona Line concept introduced by Alfaro and standardized guidelines described by Reyneke, which provide references for optimal menton positioning in relation to the lower facial third [36, 50].

Rigid internal fixation was performed using titanium miniplates and screws (Stryker^®^, Kalamazoo, Michigan, USA), following standardized osteosynthesis protocols to ensure stability of osteotomized segments. Patients were hospitalized for a median period of 1 to 3 days, depending on the complexity of the procedure and individual recovery.

Postoperative management included intravenous corticosteroids (dexamethasone 8 mg/day for 3 to 5 days) and prophylactic antibiotics (cefuroxime 1.5 g/day), followed by oral equivalents after discharge. Pain control was achieved through multimodal analgesia. During the first 72 h, patients received transdermal fentanyl (25 mcg/h), supplemented by paracetamol (1 g every 6–8 h, adjusted by body weight) and nonsteroidal anti-inflammatory drugs such as ketoprofen (100 mg every 8 h) or a combination of dexketoprofen and tramadol (25 mg/75 mg, every 8 h). Oral hydration was resumed a few hours postoperatively, and a liquid or semi-solid diet was initiated on the evening of the intervention.

The present manuscript was prepared in accordance with the STROBE (Strengthening the Reporting of Observational Studies in Epidemiology) guidelines for observational cohort studies.

Statistical analysis was performed using MedCalc Statistical Software version 20.015 (MedCalc Software Ltd, Ostend, Belgium). Non-parametric tests were used due to the non-normal distribution of satisfaction scores, as confirmed by the Shapiro–Wilk test. Group comparisons were conducted using the Mann–Whitney U test and the Kruskal–Wallis H test, with a significance level set at *p* < 0.05.

A power analysis was conducted to assess the adequacy of the sample size for the primary and secondary outcomes. Post-hoc power analysis confirmed that the comparison between patients with and without postoperative complications yielded a very large effect size (Cohen’s d = 4.14), with a power of 1.0. A prospective power analysis for gender-based differences in smile satisfaction (Cohen’s d = 0.59) revealed a power of 89.5% at α = 0.05 (two-tailed). No calculation was needed for Class II versus Class III comparison, as the mean satisfaction scores were identical (d = 0). These analyses support the robustness of the study design and the adequacy of the sample size.

## Results

Out of the 149 patients initially screened, 126 met the inclusion criteria and were enrolled in the final analysis. Of these, 75 were female and 51 were male. The average age of female patients was 21.8 years (range: 16–28), while male patients had a mean age of 22.5 years (range: 18–26). Regarding skeletal classification, 80 patients presented with Class III dentofacial deformities and 46 with Class II.

Among the 126 patients, the vast majority underwent bimaxillary procedures combining Le Fort I osteotomy with bilateral sagittal split osteotomy (*n* ≈ 120, 95.2%), while a smaller number were treated with isolated maxillary Le Fort I osteotomies (*n* ≈ 6, 4.8%). No cases required mandibular-only procedures. Additionally, six patients underwent redo surgery following previous procedures performed elsewhere. These cases were excluded from all statistical analyses and are presented only for descriptive clinical context due to their tendency to present with multiple complications.

Each participant completed the comprehensive 20-item satisfaction questionnaire at two distinct timepoints: preoperatively (for Questions 1–5, 9, and 4 regarding expected improvements) and one year postoperatively (for Questions 6–8 and 10–20). While the second assessment was performed exactly one year after surgery to ensure uniform data collection, all included patients had a minimum total follow-up duration of three years from the date of surgery.

A total of 126 patients were included in the final analysis. Of these, 14 patients (11.1%) reported postoperative complications (Table 1), while the remaining 112 patients (88.9%) reported no complications.

**Table 1:** Distribution of reported postoperative complications among the 126 patients included in the final analysis

Complication type	Frequency (*n*)	Percentage (%)
No complications	112 (88.9%)	88.9%
Infection at mandibular plate	5 (3.9%)	3.9%
Mandibular osteoperiostitis	4 (3.1%)	3.1%
Mandibular plate detachment	2 (1.6%)	1.6%
Mandibular plate fracture	1 (0.8%)	0.8%
Pseudoarthrosis	1 (0.8%)	0.8%
Malunion (vicious callus)	1 (0.8%)	0.8%
Total patients with complications	14 (11.1%)	11.1%

**Fig. 6:**
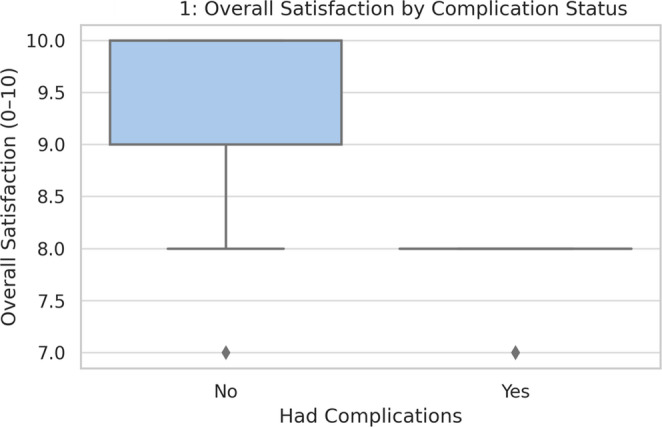
Boxplot illustrating the distribution of global satisfaction scores (0–10) in patients with and without complications

The majority of patients had no complications, while 11.1% presented adverse events of varying types (Table 1). Overall satisfaction was significantly lower in patients with complications compared to those without (Table 2), as further illustrated by the boxplot (Fig. 6). Smile-specific satisfaction remained equally high in both groups (Figure S1).

**Table 2:** Comparison of overall and smile-specific satisfaction scores between patients with and without postoperative complications

Satisfaction type	Mean (no complications)	Mean (with complications)	Mann–Whitney U	*p*-value
Smile Satisfaction	9.60	9.86	950.0	0.133
Overall Satisfaction	9.62	7.86	31.0	< 0.001

Post-hoc power analysis confirmed that the observed difference in overall satisfaction between patients with and without complications was highly significant, with a large effect size and a power of 1.0. Similarly, the comparison between male and female smile satisfaction showed sufficient statistical power (89.5%) to detect moderate differences, supporting the reliability of these subgroup analyses.

Smile satisfaction was significantly higher in female patients, while overall satisfaction did not differ by gender (Table 3). No significant differences were observed between patients with skeletal Class II and Class III deformities in terms of their satisfaction with smile aesthetics (Q8: 9.57 vs. 9.66, respectively; *p* = 0.368) or overall satisfaction with treatment outcomes (Q19: both 9.43; *p* = 0.894), indicating that the type of skeletal discrepancy did not influence subjective outcomes (Table S1).

**Table 3:** Comparison of satisfaction scores by gender

Satisfaction type	Mean (male)	Mean (female)	Mann–Whitney U	*p*-value
Smile Satisfaction	9.48	9.73	1578.0	0.025
Overall Satisfaction	9.48	9.39	2101.5	0.400

Patients who answered “Yes, definitely” to repeating surgery (Q20) had significantly higher satisfaction (mean = 9.69) than those answering “Maybe, if necessary” (mean = 7.83, *p* < 0.001; Table 4). This reinforces that high satisfaction correlates with willingness to undergo the procedure again.

**Table 4:** Overall satisfaction according to willingness to repeat orthognathic surgery (Q20)

Response	Mean satisfaction	Standard deviation	*n*
Maybe, if necessary	7.83	0.38	18
Yes, definitely	9.69	0.47	109
Kruskal-Wallis H-test	H = 59.47	p = < 0.001	

Regarding patient expectations (Q14), no statistically significant differences were observed: satisfaction was slightly higher among those whose results matched or exceeded expectations (9.48 and 9.43, respectively) compared to those less satisfied than expected (8.83; *p* = 0.100; Table S2).

Preoperatively (Q1), the most frequent motivation for surgery was facial aesthetic improvement (*n* = 86), followed by bite/mastication correction (*n* = 14), smile harmony (*n* = 9), and breathing improvement (*n* = 9). Only 4 patients cited psychological reasons. When ranking these motives (Q2), aesthetics and function were most often rated as top priorities.

Before surgery, 103 patients (81.7%) expected a major change in their appearance (Q3), and 90 expected improvements in facial profile (Q4). The majority felt well informed about risks and complications (Q5), with 115 patients (91.3%) answering “Yes.”

On a scale of 0 to 10, smile satisfaction (Q8) was remarkably high, with a mean score of 9.63 ± 0.60. Most patients (75%) scored 10, and no score was lower than 7. As for the qualitative perception of smile improvement (Q6), 103 patients (81.7%) selected “much better,” and 23 chose “slightly better.” Only one patient gave a duplicate or ambiguous entry.

Confidence while smiling in public or photos (Q7) improved notably: 105 patients (83.3%) felt “much more confident,” and 22 “slightly more confident.” No patient reported reduced confidence.

When asked about general self-confidence (Q10), 72 patients (57.1%) reported a significant improvement, and 39 (30.9%) reported a moderate improvement. Only 16 described a slight improvement, and none selected “not at all.”

Comfort in social interactions (Q11) also increased: 66 (52.4%) reported feeling “much more” comfortable, and 50 (39.7%) “slightly more.” Eleven patients reported no change.

Facial aesthetics (Q12) were rated as “very significant” by 78 patients (61.9%), “moderate” by 37, and “minor” by 11. One entry was ambiguous. Bite improvement (Q13) followed a similar pattern: 70 rated it “very significant,” 46 as “moderate,” and 11 as “minor.”

Regarding psychosocial outcomes, changes in social relationships (Q15) and professional life (Q16) did not significantly influence satisfaction. Those who reported “much better” social relations scored 9.53, and those reporting “slightly better” scored 9.30, compared to 9.45 in the “no change” group (p = 0.576; Table S3). Professionally, those who reported a positive impact scored 9.37, compared to 9.47 in the “neutral” group and 9.75 among those reporting a negative impact (p = 0.616; Table S4).

The present findings indicate that patient-reported outcomes following orthognathic surgery were highly favorable across both functional and aesthetic dimensions. Improvements in smile appearance, facial profile, self-confidence, and social comfort were widely reported. Among the variables analyzed, the presence of postoperative complications was the main observed factor associated with lower overall satisfaction. Other factors, including gender (with the exception of smile-specific perception), skeletal class, preoperative expectations, and reported psychosocial changes, showed no significant influence on long-term satisfaction.

## Discussion

The present study confirms that orthognathic surgery leads to high long-term patient satisfaction across esthetic, functional, and psychosocial domains, with a mean global score of 9.62 and a smile satisfaction of 9.63. These results are consistent with prior research emphasizing favorable subjective outcomes following bimaxillary procedures [8–47].

A key strength of this study lies in the use of a customized 20-item questionnaire that integrated validated elements from the OQLQ, DAS-59, OHIP-14, and VAS tools [51–55]. Unlike conventional assessment methods, our instrument enabled patients not only to rate their satisfaction but also to prioritize their treatment goals from a personal perspective. This structure provided deeper insight into patient concerns, revealing that facial aesthetics and smile appearance were overwhelmingly prioritized over functional improvements, confirming the central role of esthetics in patient motivation and satisfaction.

Importantly, the low number of patients who reported outcomes as “less than expected” contrasts with previous literature, where unmet expectations are more frequently cited as a source of dissatisfaction [56]. In our study, this discrepancy may be explained by three interrelated factors: the preoperative exclusion of patients with unrealistic goals or psychological contraindications, the consistent interdisciplinary involvement of both the maxillofacial surgeon and the orthodontist throughout the diagnostic and treatment process, and the deliberate avoidance of photorealistic 3D soft tissue simulation results prior to surgery. Recent studies suggest that such visualizations may generate unrealistic expectations, particularly when final outcomes are influenced by individual skeletal morphology and soft tissue variability [57, 58].

In our concept, we emphasized the chin as a central element in the architecture of facial harmony and its substantial contribution to overall satisfaction following orthognathic surgery. While other surgical disciplines, particularly plastic surgery, frequently rely on alloplastic chin implants, we adopted a different approach, favoring sliding genioplasty due to its favorable long-term predictability and biological integration. This technique, performed via an intraoral route, enables direct skeletal repositioning and avoids risks associated with synthetic implants, including visible scarring, implant mobility, or progressive anterior mandibular bone resorption [59–61]. In contrast to implants, osseous genioplasty ensures stable, long-lasting outcomes, supported by well-established data showing that soft tissue changes at the pogonion closely follow the bony movement in approximately 85% of cases [62]. We consider that part of the favorable outcomes observed in this study may be attributed to a skeletal-driven planning approach, in which bone structures were positioned to support not only cephalometric harmony but also optimal soft tissue projection and overall facial aesthetics. This concept aligns with the view that skeletal repositioning should serve as the foundation for esthetic enhancement, even if it slightly exceeds traditional cephalometric parameters. Indeed, facial attractiveness often benefits from slightly more protrusive contours than traditional cephalometric standards tend to accept [36, 63].

Smile satisfaction was rated slightly higher by female patients compared to male patients (mean scores: 9.73 vs. 9.48; *p* = 0.025), a statistically significant difference that aligns with previously reported gender-based differences in esthetic perception [64]. Interestingly, the type of skeletal discrepancy (Class II vs. Class III) and the severity of the initial deformity did not significantly affect satisfaction levels, which underscores the importance of individualized planning and execution over baseline anatomical characteristics [65].

**Fig. 7:**
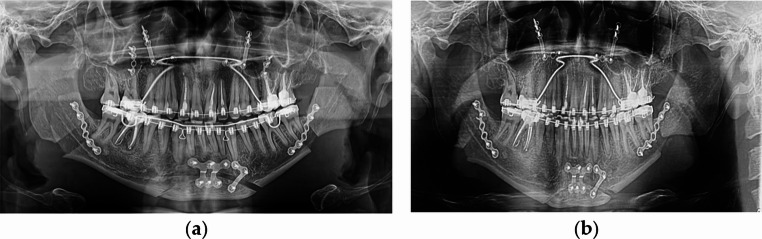
(**a**) Control radiograph immediately after surgery (**b**) Panoramic radiograph obtained 4 months later showing fracture of the right mandibular osteosynthesis plate and a persistent radiolucent gap in the healing evolution. The fractured plate remained in place with no signs of infection, and the patient was scheduled for secondary surgical management after 6 months, as only 2 months had elapsed at the time of reporting

Regarding complications, 11.1% of patients reported adverse outcomes, including infection at the mandibular plate (3.9%), mandibular osteoperiostitis (3.1%), plate detachment (1.6%), plate fracture (0.8%), pseudoarthrosis (0.8%), and malunion (0.8%) (Fig. 7).

**Fig. 8:**
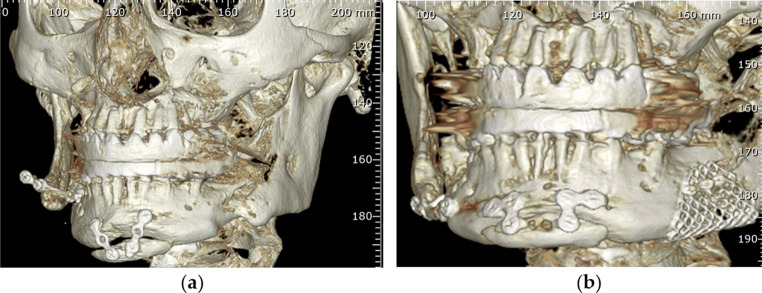
(**a**) Antero-oblique 3D-CT view of a redo case (not included in the cohort analysis) showing malunion with vicious callus, pseudoarthrosis, and partial mandibular plate detachment, associated with lateral displacement of the mandibular body, irregular bone contour, menton displacement, and a left lower border defect. (**b**) Post-redo 3D-CT scan demonstrating resolution after surgical management: replacement of the right mandibular miniplate with restored bone continuity, substitution of menton plates with L-shape miniplates and recontouring, and reconstruction of the left lower border defect using a titanium mesh fixated with screws

While malunion, pseudoarthrosis, and plate-related complications were rare in the primary cohort, redo cases referred from other centers frequently exhibited several of these events simultaneously. One such case is illustrated in Fig. 8, which demonstrates the combined occurrence of malunion with vicious callus, pseudoarthrosis, and plate detachment, all successfully managed through redo surgery.

The management of complications followed established surgical and pharmacological protocols. In cases of mandibular plate infection (*n* = 5), treatment consisted of removal of the miniplates and screws. Local curettage was performed with irrigation using 0.12% chlorhexidine gluconate solution (Corsodyl^®^, GlaxoSmithKline, UK). Systemic antibiotic therapy was administered with amoxicillin–clavulanic acid (Augmentin^®^, GlaxoSmithKline, UK), prescribed in standard adult doses of 875/125 mg orally twice daily for 7 days and adjusted according to body weight when clinically indicated. Mandibular osteoperiostitis (*n* = 4) was managed conservatively with the same systemic antibiotic regimen, and gradual remission was achieved over a 2-month period. Plate detachment (*n* = 2) was managed through surgical reintervention with replacement of the fixation plate in order to restore bone continuity. A single case of plate fracture (*n* = 1) remained clinically stable and free of infection. The fractured plate was left in place, and secondary surgery was scheduled after 6 months, as only 2 months had elapsed at the time of reporting. Pseudoarthrosis (*n* = 1) resulted from displacement and loss of contact between bone segments and was corrected by surgical reintervention with placement of a new plate. Malunion with vicious callus (*n* = 1) was also treated by reoperation with plate replacement to reestablish proper alignment and stability. All complications resolved without major sequelae, with infection cases showing remission within 2 months and functional stability being restored following surgical reinterventions.

These rates fall within the expected range of postoperative complications cited in the literature [49, 66, 67]. Notably, patients who experienced complications reported significantly lower overall satisfaction (mean = 7.86) compared to those without complications (mean = 9.62, *p* < 0.001), although smile-specific satisfaction remained stable across both groups (9.86 vs. 9.60, *p* = 0.133), indicating that functional or healing-related issues had a greater impact on global perceptions than esthetic outcomes. These findings confirm the considerable impact that postoperative complications have on patient-reported quality of life [68]. The low rates of pseudoarthrosis and malunion (each 0.8%) likely reflect the consistent use of rigid fixation with titanium miniplates and strict adherence to osteosynthesis protocols, which have been shown to reduce instability and delayed healing [69].

In addition to the long-term outcomes, observations made during postoperative consultations provided valuable insights into the early recovery period. Most patients experienced moderate postoperative pain, with discomfort more frequently attributed to nasotracheal intubation rather than the surgical procedures themselves. Temporary difficulties related to diet were also commonly reported, as patients were required to follow a soft or semi-liquid diet for at least three weeks postoperatively. Furthermore, some reported challenges in adapting to occlusal function, particularly in gauging bite strength or pressure, which gradually improved during follow-up. These minor complaints, although self-limiting, highlight the importance of preoperative counseling regarding postoperative expectations and recovery trajectory.

Although many patients reported improved social or professional life after surgery, these changes did not significantly correlate with satisfaction scores in our cohort. This contrasts with previous studies suggesting greater social reintegration after orthognathic treatment, particularly in younger or socially marginalized patients [70]. One possible explanation lies in the demographic profile of our sample, which consisted mostly of socially integrated young adults.

This study offers new insights into the long-term psychosocial benefits of orthognathic surgery by employing a multi-domain, patient-centered evaluation tool and by emphasizing the centrality of esthetic motivation. The combination of structured interdisciplinary collaboration and careful patient selection appears to be essential not only for reducing complication rates but also for optimizing long-term satisfaction.

This study has several limitations. Redo cases were excluded from the statistical analysis because they typically presented multiple complications, which could have biased the overall outcomes. Although the single-center design and the involvement of the same surgical and orthodontic team ensured consistency and minimized variability, these factors may limit the generalizability of the findings. The preoperative exclusion of patients with unrealistic expectations or psychological profiles incompatible with the goals of orthognathic surgery may also have contributed to the high satisfaction scores observed, introducing a potential selection bias. In addition, the distribution of satisfaction scores, with many patients reporting very high values, suggests a possible ceiling effect that may reduce sensitivity in detecting subtle differences between subgroups. The customized questionnaire, although developed from validated instruments, has not yet undergone large-scale external validation, and patient-reported outcomes were assessed at a fixed one-year timepoint, even though perceptions may evolve during longer follow-up. Future research should include multicenter studies with larger and more diverse cohorts, repeated long-term assessments at multiple intervals, and integration of objective functional and three-dimensional soft tissue analyses to complement patient-reported measures.

## Conclusions

This study demonstrates that orthognathic surgery achieves high long-term patient satisfaction, particularly in esthetic and psychosocial dimensions. The customized 20-item questionnaire allowed patients to prioritize treatment goals and confirmed that improvements in smile appearance and self-confidence were most appreciated. Although 11.1% of patients experienced postoperative complications, these were the main observed factor associated with lower overall satisfaction. Smile satisfaction remained high regardless of complications, gender, or skeletal class, emphasizing the importance of individualized planning and consistent interdisciplinary care. These findings support the long-term value of orthognathic surgery not only for correcting dentofacial deformities, but also for enhancing quality of life through patient-centered outcomes. 

## Supplementary Information

Below is the link to the electronic supplementary material.

Supplementary Material 1 The following supporting information can be found at the article's webpage on the publisher’s site: Figure S1: Smile Satisfaction by Complication Status; Table S1: Comparison of Satisfaction Scores Based on Orthognathic Class; Table S2: Overall Satisfaction According to Patient Expectations (Q14); Table S3: Overall Satisfaction According to Social Relationship Changes (Q15); Table S4: Overall Satisfaction According to Professional Life Impact (Q16).

## Data Availability

The data supporting the findings of this study are not publicly available due to patient privacy and ethical restrictions.

## Abbreviations

TMJ: Temporomandibular Joint
OQLQ: Orthognathic Quality of Life Questionnaire
DAS-59: Derriford Appearance Scale-59
OHIP-14: Oral Health Impact Profile-14
VAS: Visual Analogue Scale
BSSO: Bilateral Sagittal Split Osteotomy
SARPE: Surgically Assisted Rapid Palatal Expansion
DFD: Dentofacial Deformity
CT: Computed Tomography
3D-CT: Three-Dimensional Computed Tomography
STROBE: Strengthening the Reporting of Observational Studies in Epidemiology
GDPR: General Data Protection Regulation
CAD/CAM: Computer-Aided Design / Computer-Aided Manufacturing
