# Supplementary material for: Long-term patient satisfaction and complication rates after orthognathic surgery: a minimum 3-year follow-up study
Source: Oral Maxillofac Surg. 2026 May 27;30(1):92. doi: 10.1007/s10006-026-01580-2 (PMC13212630; doi:10.1007/s10006-026-01580-2)
Supplement: Supplementary file 1 — Supplementary Material 1 The following supporting information can be found at the article's webpage on the publisher’s site: Figure S1: Smile Satisfaction by Complication Status; Table S1: Comparison of Satisfaction Scores Based on Orthognathic Class; Table S2: Overall Satisfaction According to Patient Expectations (Q14); Table S3: Overall Satisfaction According to Social Relationship Changes (Q15); Table S4: Overall Satisfaction According to Professional Life Impact (Q16). [file 10006_2026_1580_MOESM1_ESM.docx]

# Figure S1: Smile Satisfaction by Complication Status


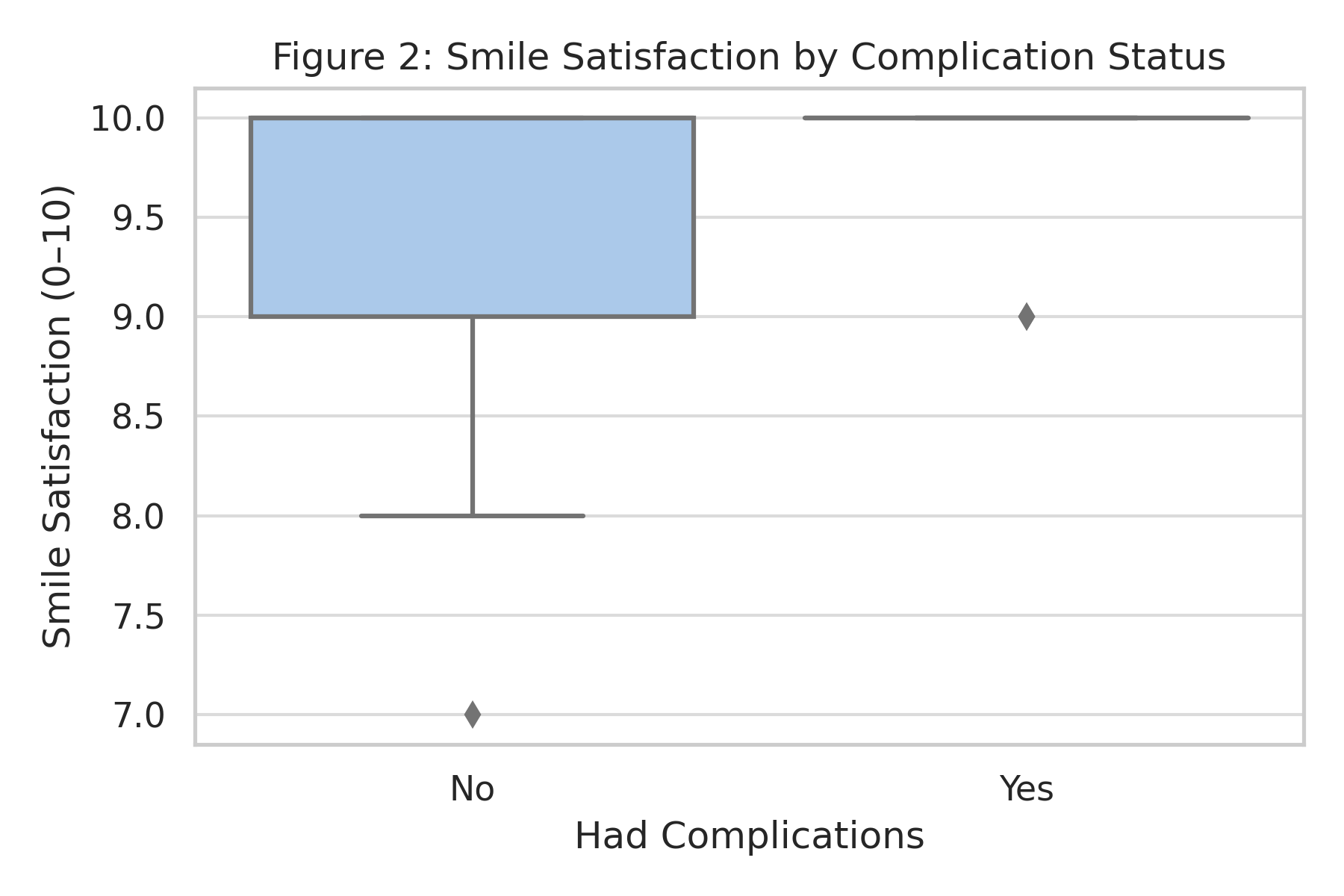


Boxplot showing the distribution of smile satisfaction scores (0–10) between patients with and without postoperative complications.

# Table S1: Comparison of Satisfaction Scores Based on Orthognathic Class

| Satisfaction Type | Mean (Class II) | Mean (Class III) | Mann–Whitney U | p-value |
| --- | --- | --- | --- | --- |
| Smile Satisfaction | 9.57 | 9.66 | 1733.0 | 0.368 |
| Overall Satisfaction | 9.43 | 9.43 | 1856.0 | 0.894 |

# Table S2: Overall Satisfaction According to Patient Expectations (Q14)

| Expectation Group | Mean Satisfaction | Standard Deviation | n |
| --- | --- | --- | --- |
| As expected | 9.48 | 0.70 | 61 |
| Better than expected | 9.43 | 0.87 | 60 |
| Less than expected | 8.83 | 0.75 | 6 |
| Kruskal-Wallis H-test | H = 4.61 | p = 0.100 |  |

# Table S 3: Overall Satisfaction According to Social Relationship Changes (Q15)

| Response | Mean Satisfaction | Standard Deviation | n |
| --- | --- | --- | --- |
| No change | 9.45 | 0.60 | 20 |
| Slightly better | 9.30 | 0.95 | 50 |
| Yes, much better | 9.53 | 0.68 | 57 |
| Kruskal-Wallis H-test | H = 1.10 | p = 0.576 |  |

# Table S 4: Overall Satisfaction According to Professional Life Impact (Q16)

| Response | Mean Satisfaction | Standard Deviation | n |
| --- | --- | --- | --- |
| Negatively | 9.75 | 0.50 | 4 |
| Neutral | 10.00 | nan | 1 |
| Neutral | 9.47 | 0.78 | 51 |
| Yes, positively | 9.37 | 0.81 | 71 |
| Kruskal-Wallis H-test | H = 1.79 | p = 0.616 |  |
